# Acceptability of peer learning and education approach on malaria prevention (PLEA-malaria) through primary schools communities in rural Ethiopia: peer educators’ perspectives

**DOI:** 10.1186/s12936-021-03965-y

**Published:** 2021-11-15

**Authors:** Fira Abamecha, Alemayehu Deressa, Morankar Sudhakar, Lakew Abebe, Yohannes Kebede, Dejene Tilahun, Firanbon Teshome, Zewdie Birhanu

**Affiliations:** 1 Department of Health, Behavior and Society, Faculty of Public Health, Institute of Health, Jimma University, Jimma, Ethiopia; 2 Department of Public Health and Policy, College of Health and Medical Science, Haramaya University, Harar, Ethiopia

**Keywords:** Peer education, SBCC, Malaria, Acceptability, School, Ethiopia

## Abstract

**Background:**

Evidence on peer educators’ experiences of implementing the school-based educational interventions on malaria prevention would be used as inputs for malaria eliminating efforts. This study explored the acceptability of the school-based peer-learning and education approach on malaria prevention (PLEA-malaria) among peer educators in Ethiopia.

**Methods:**

This process evaluation study was aimed to examine the success of the school-based PLEA-malaria that was implemented in 75 primary schools in Jimma from 2017 to 2019. A mixed research method was employed to collect post-intervention data from 404 peer educators and key stakeholders. Data were collected using a structured questionnaire and interview guide. Multivariable linear regression modelling was performed using SPSS software version 26.0. Atlas ti 7.5 for windows was used to analyse the qualitative data. The result was presented by triangulating the findings of the qualitative and quantitative methods.

**Results:**

The mean score (M, range = R) of acceptability of PLEA-malaria was (M = 20.20, R = 6–30). The regression modelling showed that age; (β = 0.264, 95% CI 0.266 to 0.632), GPA; (β = 0.106, 95% CI 0.008 to 0.074), parental readiness for malaria education; (β = 0.184, 95% CI 0.711 to 2.130), frequency of peer education; (β = 0.232, 95% CI 1.087 to 2.514) and team spirit; (β = 0.141, 95% CI 0.027 to 0.177) were positively associated with the acceptability while this relationship was negative for the number of ITN in the household; (β =  − 0.111, 95% CI − 1.182 to -0.13) and frequency of parent-student communication; (β =  − 0.149, 95% CI  − 1.201 to − 0.293). The qualitative study identified facilitators of PLEA-malaria (e.g. team formation process, outcome efficacy, presence of schools’ structures, schools priority, and support) and barriers (e.g. low commitments, threat appraisal, response efficacy, and PLEA-malaria implementation gaps).

**Conclusion:**

The results suggested that the acceptability of the school-based PLEA-malaria was higher implying the strategy is promising in promoting malaria prevention in primary schools. Considering factors related to personal, access to malaria preventive services, school system, and social support in education and behaviour change interventions would be important to improve the acceptability. The relationship about how an improvement in the level of acceptability would in turn influences malaria preventive behaviours among the students should be investigated.

**Supplementary Information:**

The online version contains supplementary material available at 10.1186/s12936-021-03965-y.

## Background

With nearly, half of the global population being at risk, malaria continues to be the major social security and economic threat [1]. Malaria is one of the vector-borne diseases transmitted by the bite of the infected *Anopheles* mosquito [2]. The world health organization (WHO) reported that the estimate of global malaria cases in 2019 was 229 million with 409,000 lives lost [3]. More than 90% of malaria deaths occur in sub-Saharan Africa [3]. Ethiopia is one of the African countries with 52% of its population is at risk for malaria [4]. Although it affects every member of the community, malaria has profound health and social effects on school-aged children affecting the critical period of learning and development [5, 6].

The world is now committed to an ambitious goal for malaria elimination by 2040–2050 using cost-effective participatory public health interventions [7–9]. Promoting essential preventive measures such as vector control, including insecticide-treated net (ITN) and insecticide residual spray (IRS), diagnosis and case management and ecological or breeding sites management were important to accelerate the malaria elimination goal [8]. Moreover, Ethiopia is one of the African countries that adopted the ambitious malaria elimination goal by 2030 and beyond by promoting the participation of the community and community-based institutions such as schools [4]. The cost-effective strategies involve diverse approaches such as interpersonal communication (IPC) or peer-led education, institutional and community engagement in malaria preventive programmes [10, 11]. Engagement of schools communities (students, teachers, schools principals) and community health workers in health behaviour change efforts is believed to magnify the outcomes.

Embracing the child-centred and child-empowering values through meaningful participation would provide them an opportunity to express their interests and play their active roles as recognized part of the public [12]. In addition, to elevating the participatory rights of the children; engaging children in innovative programmes and research projects would help the success of the programme [12]. Enabling and encouraging children to articulate their own perspectives and experiences about an issue that matters would help to achieve real benefits to them and their communities [12]. Key to these facts, the participatory behaviour change and health promotion interventions in schools provide an opportunity for implementing student-centred school-based peer education.

The peer learning and educational approach on malaria prevention (PLEA-malaria) is defined as a system of teaching or sharing of information, values, and behaviours about health issues by members of similar age or status to improve social learning and provide psychosocial support [13]. Peer learning is a potentially powerful way of sharing knowledge and experiences among peers, and potentially diffusing this learning back to their organizations to ensure a positive impact [14]. Empirical evidence showed that trained school children served as change agents by addressing their peers and the local community with health education on malaria prevention and control practices [15]. Another, evidence showed deploying schools using students as vehicles demonstrated proven effects to addressing numerous health-related problems including Water, Sanitation, and Hygiene (WASH), HIV/AIDS, and malaria prevention in the community [16–18]. The successful implementation and sustainment of malaria preventive peer education through school depends on the attitude and acceptability of the students, peer educators, and other school communities.

However, there is no evidence in the scientific literature on experiences and acceptability of school-based PLEA as far as malaria prevention is concerned. Thus, exploring the perceptions and experiences of peer educators on the school-engaged PLEA has paramount importance to understanding the mechanism by which students are engaged in promoting malaria prevention in schools and local communities. Enrolling peer educators; this study examined the experiences and acceptability of PLEA on malaria prevention and control practices in primary schools in malaria-endemic rural Ethiopia.

## Methods

### Study setting

The current study is a part of the several studies [19–23] done to evaluate the effectiveness of the school-based malaria social and behaviour change communication (SBCC) interventions on advancing malaria prevention and control practices in primary schools in Jimma, Ethiopia. The school-based SBCC was implemented in 75 schools that are found in five districts of Jimma from 2017 to 2019. The aim of the programme was to promote malaria prevention and control actions in schools and villages where malaria transmission rates were relatively high [19–23].

### The school-based malaria SBCC approach

A participatory approach was followed to develop and implement the current school-based SBCC intervention aimed to advance community practice on malaria prevention and control actions in 75 primary schools and respective villages. The school-based PLEA-malaria (which was the main area of concern of the current study) is one of the major components of the school-based malaria SBCC strategy that was implemented in selected schools. Accordingly, the development of the SBCC programme was informed by evidence from formative qualitative research conducted in the area to explore the local malaria situation, community needs and to map the key stakeholders in the area. Various data collection techniques including in-depth interviews (IDIs) and documents review were employed to collect rich data. A review of documents of the routine health care on malaria incidence in all districts of Jimma Zone was done to examine the malaria incidence rate based on the estimates of annual parasite incidence (API) rates. Thus, the five districts of Jimma Zone targeted by the current programme were selected based on the API rate and access to malaria preventive services in the districts.

Face-to-face IDIs were conducted with purposively selected individuals or stakeholders from health departments, education sectors, and schools. This includes the heads of health departments, heads of education offices, school directors, selected school teachers, malaria focal persons in health departments, and the health extension workers (HEWs). The purpose was to explore malaria-related beliefs, threat perceptions, preventive practices, opportunities, organizational appropriateness, and barriers for malaria preventive practices in the community and schools. Moreover, issues about the existing primary health care (PHC) practices related to malaria, school systems, and inter-sectoral activities (between schools, health departments) were explored. The findings of the qualitative research were then used as the baseline evidence to design the school-based malaria SBCC strategy and packages (“participant enrichment”) and to design the subsequent quantitative research instruments (“instrument validity”) that was used in the end-line evaluation studies.

The implementation current programme was guided by the SBCC strategy that involves diverse approaches including participatory capacity building (e.g. training), building/strengthening systems, and interpersonal communication activities (e.g. PLEA-malaria) with aim of promoting sustained malaria preventive actions both at schools and in villages. It was implemented from 2017 to 2019 in 75 primary schools and respective rural villages where malaria transition was endemic. The ultimate goal of the programme was to empower schools, local stakeholders and the community to cooperatively design, implement and evaluate the key malaria preventive actions that include sustained use of ITNs, appropriate & timely seeking care for malaria, appropriate use of anti-malaria drugs, acceptance of IRS, and mosquito breeding habitat modifications (e.g. gardening and solid waste management) were done in the schools and villages.

The school-based SBCC strategy was mainly situated in primary schools that employed several hierarchies (step-by-step process) to reach the rural residents and school communities with malaria preventive messages and recommended actions. Accordingly, the first-level basic training on malaria, malaria prevention and control, community mobilization, and basic premises of the SBCC strategy was given for the key stakeholders such as heads of and deputy heads of the health departments and education offices, malaria focal persons, school directors, selected school teachers and HEWs. Following completion of the training, participants were oriented and assigned their roles and responsibilities in each programme component and activity (Additional file 1). For instance, the health department; in collaboration with the education office; was given primary roles of monitoring and supervision of the school-based malaria education and communication. While roles of initiating, guiding, and supervising the school-based PLEA-malaria were given for schools and teachers under close supervision of school directors. All the health departments, education offices, and schools are accountable to the field coordinators who were in turn accountable to the main office of the SBCC programme. The pieces of training, monitoring, and supervision of the implementation were accomplished by six full-time field officers assigned to the five districts.

The trained school directors and teachers (under supervision by the field officers); were supported and supervised to cascade the second-level training down to the school peer educators selected from the circle of students (locally called Geengoo barattootaa in Afan Oromoo). The circle of students is a group-based peer education approach in which up to six individual students (1 leader and 5 members in one group) sit in a circle to promote group-based learning activities aimed to improve academic performance. The purpose is to promote supports among students through a peer learning network. The third-level intervention involved planning, implementing, and monitoring regular school-based PLEA-malaria activities enrolling all school students. The school-based PLEA-malaria was facilitated by trained peer educators under the guidance of the school teachers and school directors. The frequency or schedules for PLEA-malaria were developed autonomously by the schools based on the existing circumstances in each school such as the nature of school policy or programmes.

Accordingly, different schools implemented different schedules that range from every week to every three weeks, with the majority being every two weeks. The aim of school-based PLEA-malaria was to empower the students on malaria prevention and enable them to act as health agents or health messengers to disseminate malaria preventive information to parents and neighbours. All schools students were reached by the school-based malaria education programme and took a post-test before they were sent to teach their parents, neighbors, and community members. The peer education activities were aided by manuals and various health learning materials (HLMs) such as flip charts, leaflets, and posters with persuasive messages. Further, various educational and communication activities such as social dramas, campaigns, and role-plays were conducted within schools and in the nearby communities.

At the fourth level, all school students equipped with malaria knowledge and skills on its prevention were instructed to reach out to their parents and neighbors with malaria messages and various outreach activities. This process of training the peer educators who will, in turn, implement or guide the peer learning activities for the whole school students on malaria before they were sent out to educate or disseminate the messages to parents and the local community was conceptualized in this intervention as the PLEA-malaria. Rigorous supportive supervision, field visits, and review meetings were implemented in participatory ways to build sustainability and ownership of the SBCC activities. The school-based malaria SBCC/PLEA-malaria was integrated into the existing primary health care activities in which the HEWs were actively collaborated to cascade it to the household level through women-centered health development teams (HDT) and larger community groups to ensure long-lasting sustainability. Details about the school-based SBCC and PLEA-malaria were presented in previous studies published elsewhere [19–23].

### Study design and evaluation outcomes

A parallel mixed method was conducted from April to June 2020 to collect post-intervention data on the experiences and acceptability of the school-based PLEA-malaria in schools targeted by the programme. The study mainly explored the peer educators’ experiences, perceptions, and acceptability of the school-based PLEA-malaria and associated barriers and facilitators to implementing the programme. The quantitative method was employed to measure the level of acceptability of the PLEA-malaria. The complementary qualitative study was conducted in a broader context to explore the experiences and perspectives of the participants (key stakeholders) on the implementation process, facilitators, and barriers [24].

### Study population and sample

#### Quantitative method

The study enrolled peer educators who have been leading and providing malaria education to their peers and parents over the programme period. There were about 8842 trained peer educators in 75 primary schools that were reached by the programme. They received basic training on malaria causes, signs and symptoms, prevention and control, and basic skills of guiding and running the PLEA both in schools and the community. The sample size was determined by using a single population proportion formula as follows: $n=(Z\mathrm{\alpha }/2)2\frac{P(1-P)}{d2}$**,** Where, n = desired sample size, P = 0.5 estimated variability in scores of acceptability of PLEA-malaria among the peer educators. It takes on value (i.e. 0.5) which yields a maximum sample size. Alpha, α = 0.5: confidence interval at 95%, d = 0.05: desired precision or margin of error. This gives a sample size of 384. After correcting for the total population of 8,842 and adding 10% for non-response rate, gives a total sample of 404.

#### Sampling procedure

There were total populations of 8842 peer educators in 75 primary schools from grade 6th through 8th. Approximately, there were about 118 peer educators in each school (i.e. 8842/75 = 118). About 15 schools were randomly selected with about 5310 participants (i.e. 15*118 = 5310). In order to draw a sample size of 404 from 15 schools, approximately 27 peer educators have to be selected from each school with nine (9) participants from each grade. Data were collected by face-to-face interview with participants drawn from grade 6^th^ through grade 8^th^ in 15 primary schools.

#### Data collection tool and procedure

Data were collected using a structured questionnaire designed for addressing the socio-demographic factors, the intervention outcomes, perceptions, and peer education experiences were adapted from related studies [25–28]. The questionnaires were prepared in English and translated into *Afan Oromo*, the local language. Trained interviewers have collected the data from the selected participants in quite a space prepared in the schools. Data were checked for completeness and consistency after each day of data collection by supervisors.

#### Measurements

Knowledge: Multiple questions were used to tap the malaria-related knowledge including the causes, symptoms, methods of prevention, and timing of vector biting behaviours [29]. It was captured using nine (9) items such that Yes = 1 for correct statements and No = 0 for incorrect statements. The scores of correct answers were computed and a higher score was interpreted as a higher level of knowledge on malaria.

Attitude: Attitude was defined as individuals’ affective feelings towards the various forms of control practices including the mosquito nets and their use, malaria treatment-seeking, use of anti-malarial medicines, and IRS. A question consisting of six items (α = 0.76) was designed in a 5-point Likert scale format ranging from 1 = strongly disagree to 5 = strongly agree. The higher composite score was interpreted as a positive or supportive attitude.

Perceived susceptibility of malaria: the perceived susceptibility construct was conceptualized in this study as beliefs about the likelihood of experiencing or acquiring malaria given that the local situation. This dimension was explored by using three items (α = 0.73) designed on a 5-point Likert scale format such that 1 = strongly disagree to 5 = strongly agree. Responses were added up to create a composite score that was interpreted as a higher score representing higher beliefs of susceptibility.

Perceived severity: The perceived severity of malaria was an individual’s perception that malaria leads to bad health and social outcomes that could interfere with their routine jobs (e.g. attending school, and school performances [21]. The perceived severity of malaria was measured using questions consisting of four items (α = 0.76) constructed on a 5-point Likert scale format with 1 = strongly disagree to 5 = strongly agree. The negatively worded items were reverse-scored before creating the composite score. A higher composite score was interpreted as higher beliefs of severity.

Parent and student communication practices: This component encompasses the beliefs about parental readiness or acceptance of malaria education by children, the frequency of parent and student discussion on malaria and its preventive measures (e.g. about ITNs, IRS, treatment-seeking for fever, compliance to anti-malaria medicines/drugs) in the last 12 months. One item each was used to measure: (1) the parent and student communication practices using Yes = 1, if they ever practiced and otherwise, No = 0, (2) parental readiness/acceptance of the malaria education using low = 1, moderate = 2 and high/good = 3 and (3) frequency of the parent and student communication using rarely = 1, occasionally = 2 and always = 3.

Malaria preventive practices: This includes various malaria preventive measures that were undertaken by peer educators such as access to ITN, using the ITNs every night and time to healthcare-seeking behaviours for fever. Specifically, the time of healthcare-seeking behaviors was measured in 24 h formats for appropriate interpretation [30].

Self-efficacy towards the PLEA-malaria: This dimension explored the peer educators’ perceived confidence or self-efficacy of implementing or running the PLEA-malaria [27, 31]. Five items (α = 0.83) were used to measure this construct on a 5-point Likert scale. The responses were summed up to form composite scores in which high scores indicated high self-efficacy.

Team spirit: This was conceptualized as the perceptions of the participants about the team’s ability, collective commitments, and trust to share knowledge and experiences to improve the performance of the team towards their common goal [32]. Nine items (α = 0.84) designed using the 5-point Likert scale were used to measure this component. Responses were summed up to form scores with a high value indicating high perceptions of team spirit.

Acceptability of an intervention: This construct was considered the dependent variable of the current study that explored the peer educators’ perceptions that the PLEA-malaria is appealing or beneficial to them and to others in terms of expectations, preferences, felt needs regarding the malaria situation [33, 34]. Six items (α = 0.81) were adapted from a previous study [35] and designed to capture the perceptions of peer educators about the benefits of the PLEA-malaria in addressing malaria problems in the area. Items were constructed by using a 5-point Likert scale format such that 1 = strongly disagree to 5 = strongly agree. Responses were added up to form a composite score that was interpreted as a higher value of the score was considered the acceptability of the PLEA-malaria.

### Quantitative data analyses

The data were analysed using the statistical packages for social sciences (SPSS) 26.0 software for windows. Descriptive statistical measures such as frequency, mean, proportion, and standard deviation were computed. The Pearson’s correlation analysis was carried out to examine the relationship between the acceptability of the school-based PLEA-malaria and other psychosocial variables as bivariate analysis. Similarly, an independent sample t-test was carried out to explore associations between acceptability and other dichotomous variables.

Multivariable linear regression modelling was performed to identify the factors associated with the acceptability of the school-based PLEA-malaria. Standardized regression coefficients (beta) were interpreted to understand the independent effects of the selected predictors on the outcome variable. A P-value less than 5% were considered for the significant association.

### Qualitative methods

#### Participants

The participants were selected based on prior knowledge about the programme experiences, exposure to the interventions and contexts [24]. The experiences and perspectives of participants that include school directors, public health officials, students and teachers) on the programme implementation and adoption process were explored. Thus, the purposive sampling technique was employed to recruit informants who have actively engaged in the programme as the leading stakeholders and peer educators. Various segments of the stakeholders, teachers and students were reached out with the ultimate goal of maintaining maximum variability sampling assumptions [36]. The intent was to address multiple perspectives, experiences, and factors influencing the programme implementation and effectiveness. Accordingly, in-depth interviews (IDIs) were conducted with purposively selected teachers, school directors, public health officials and students who had key administration roles in the programme.

#### Qualitative data collection and analysis

In-depth interviews were conducted with the selected participants using an interview guide that was developed based on the contents and propositions of the quantitative study. The interviews were conducted in quiet spaces found in the back yard of each school and some interviews were conducted in offices/quite rooms in the schools. Data collection was assisted by audiotape records and the data collectors took field notes. The average recorded time to run the KIIs was 45 min.

#### Qualitative data analysis

The recordings and field notes were transcribed verbatim and translated into English. The Atlas. ti 7.1 software for analysis was used to guide the analysis process. Data analysis was done through the thematic analysis process to build conceptually defined themes [36]. Initial analysis was started with quick identification and assigning of codes to descriptive data segments based on pre-defined research questions or prepositions identified in research objectives. Further coding was done to clear out any relevant emerging segments that require assigning new codes and alternative explanations. Descriptive and conceptual definitions were given to each code and emerging concepts through appropriate interpretations. Coding was done independently by two experienced public health researchers. Besides, quotes of participants’ expressions that exemplify key concepts were used directly during analysis and interpretation. Finally, the results and discussion are presented by triangulating the qualitative and quantitative findings.

## Results

### Socio demographic characteristics

A total of 401 out of 404 peer educators have participated in this study. Among the total participants, the majority, 60.3% (242) were male and age group between 15 and 19 years, 57.1% (229) with a mean age of 15.59 (± SD 2.24) years. The majority of participants, 73.6% (295) and 85.8% (344) were Muslims and Oromo ethnic groups respectively. About 68.6% (275) of the participants have no leadership roles in the class or were class members (Table 1).

**Table 1:** Socio-demographic characteristics of the quantitative study participants targeted by the school-based PLEA-malaria, Jimma, Ethiopia, 2017–2019 (N = 404)

Categories	Frequency	Percentage (%)
Place of residence		
Urban	61	15.2
Rural	340	84.8
Sex		
Male	242	60.3
Female	159	39.7
Age of students		
10–14	144	35.9
15–19	229	57.1
20–24	28	7.0
Religion		
Muslim	295	73.6
Orthodox	72	18.0
Protestant	34	8.5
Ethnicity		
Oromo	344	85.8
Amhara	31	7.7
Others	26	6.4
Roles in class		
Class leader	69	17.2
Vice leader	57	14.2
Members	275	68.6
Latest GPA		
Excellent	48	12.0
Very good	155	38.7
Satisfactory	182	45.4
Fair	16	4

### Malaria knowledge and preventive behaviours

Various peer education and learning activities were conducted in the schools in the two programme period. According, the majority 66.9% (228) of the study groups reported that they had conducted peer education in school every two weeks above. Various topics of the PLEA-malaria were mentioned as major points of discussion at peer education sessions that include issues related to ITN use and handling, 43.4% (174), prompt care-seeking practices for fever, 25.9% (104) and anti-malarial drugs use, 27.7% (111) and environmental sanitation, 37.4% (150) were reported.

Considerably the highest number of the study participants, 90.8% (364) reported having at least one ITN in the household. Most participants, 57.4% (230), have reported having 2–3 ITNs in the household. The highest proportion of the study participants, 92.3% (224) were found using ITN every night. Out of 8.2% (33) of the participants who have experienced fever in the last two weeks, all most all; 7.7% (31) have sought health care for the fever. However, only 4.7% (19) of the participants sought health care before 24 h after the fever has occurred (Table 2).

**Table 2:** Peer education activities and malaria preventive behaviours among the peer educators in primary schools targeted by the PLEA-malaria, Jimma, Ethiopia, 2017–2019, (N = 404)

Variables	Frequency	Percentage
Frequency of school-based peer education		
Every weeks	173	43.2
Every 2 weeks and above	228	66.9
Specific topics of the PLEA-malaria		
About ITN use and care/handling	174	43.4
About prompt care seeking for fever	104	25.9
About anti-malarial drugs	111	27.7
About environmental sanitation	150	37.4
Malaria preventive behaviours		
Have at least one ITN in the house hold		
Yes	364	90.8
No	37	9.2
Number of ITN in household		
= 1	77	19.2
2–3	230	57.4
4+	94	23.4
ITN utilization		
Yes	224	92.3
No	140	34.9
Experienced fever in the last 2 weeks		
Yes	33	8.2
No	368	91.8
Sought care for the fever (n = 33)		
Yes	32	7.7
No	1	0.5
Prompt care seeking behaviours (n = 33)		
After 24 h	14	3.5
Before 24 h	19	4.7

### Descriptive and Pearson correlation (*r*) parameters

The result showed that the score of acceptability of PLEA-malaria was considerably high or above the mean value with a score of 20.20 (SD = 3.86). Except for knowledge and attitude; which showed lower mean scores; 9.16 (SD = 3.42) and 9.43 (4.34) respectively; all the other psychometrically measured constructs have higher mean scores. Except for knowledge and attitude; all the remaining constructs were significantly correlated with the acceptability score. The perceived susceptibility to malaria was negatively correlated with the acceptability and all the other constructs positively correlated with it. The lowest and highest correlation with acceptability score was that of perceived risk; r = − 0.134, p < 0.01 and self-efficacy; r = 0.334, p < 0.01 respectively (Table 3).

**Table 3:** Descriptive and Pearson’s correlation parameters for the measures of psychosocial among peer educators in primary schools, Jimma, Ethiopia, 2017–2019, (N = 404)

	Variables	1	2	3	4	5	6	7
1	Acceptability	–						
2	Knowledge	0.082	–					
3	Attitude	− 0.035	− 0.115^*^	-				
4	Perceived severity	0.170^**^	0.089	0.011	−			
5	Perceived susceptibility	− 0.134^**^	0.060	− 0.465^**^	− 0.261^**^	−		
6	Team spirit	0.302^**^	0.098^*^	− 0.135^**^	0.240^**^	− 0.085	−	
7	Self-efficacy	0.334^**^	0.175^**^	− 0.088	0.299^**^	− 0.102^*^	0.403^**^	-
	Number of items	6	30	6	4	3	9	5
	Scale range	30	0–30	6–30	4–20	3–15	9–45	5–25
	Mean score	20.20	9.16	9.43	16.30	9.27	37.18	24.97
	Standard deviation	3.86	3.42	4.34	2.30	3.22	5.33	3.29

^*^. Correlation is significant at the 0.05 level (2-tailed), **. Correlation is significant at the 0.01 level (2-tailed)

### Independent predictors of acceptability of the PLEA-malaria

Multivariable linear regression modelling was performed with 15 selected variables such as age in years, grade level, ethnicity, GPA, number of ITN in the household, ITN use, frequency of parent and student communication, parental readiness towards malaria education, team spirit, satisfaction with team formation process, perceived risk, perceived severity, attitude, knowledge and self-efficacy. However, the result indicated only age, number of ITN in the household, GPA, frequency of parent and student communication, parental readiness for malaria education, frequency of peer education, team spirit, and self-efficacy were emerged out affecting the acceptability of the PLEA-malaria at p < 0.05.

Accordingly, age in complete years; (standardized β = 0.264, 95% CI (0.266 to 0.632), GPA; (standardized β = 0.106, 95% CI (0.008 to 0.074), parental readiness for malaria education; (standardized β = 0.184, 95% CI (0.711 to 2.130), frequency of peer education; (standardized β = 0.232, 95% CI (1.087 to 2.514) and peer education team spirit; (standardized β = 0.141, 95% CI (0.027 to 0.177) positively associated with acceptability of the school-based PLEA-malaria. However, some factors such as number of ITN in the household; (standardized β = − 0.111, 95% CI (− 1.182 to − 0.13) and frequency of parent and student communication; (standardized β = − 0.149, 95% CI − 1.201 to − 0.293) negatively predicted the acceptability of PLEA-malaria (Table 4).

**Table 4:** Multivariable linear regression modelling parameters for the acceptability of school-based PLEA-malaria among peer educators in primary schools, Jimma, Ethiopia, 2017–2019, (N = 404)

Variables	Unstandardized coeffs. (β)	Standardized coeffs. (β)	95% CI for β	p-value
Age in years	0.449	0.264	(0.266, 0.632)	0.000
Gender	0.545	0.069	(− 0.131, 1.220)	0.114
Grade level	0.215	0.045	(− 0.207, 0.637)	0.318
GPA (latest semester)	0.041	0.106	(0.008, 0.074)	0.015
Religion	− 0.466	− 0.076	(− 1.062, 0.130	0.125
Ethnicity	0.378	0.053	(− 0.325, 1.080)	0.291
Frequency of parent student and communication	− 0.747	− 0.149	(− 1.201, − 0.293)	0.001
Frequency of peer education	1.801	0.232	(1.087, 2.514)	0.000
Parental readiness to malaria education	1.420	0.184	(0.711, 2.130)	0.000
Number of ITN in the household	− 0.658	− 0.111	(− 1.182, − 0.135)	0.014
ITN utilization	0.529	0.066	(− 0.196, 1.255)	0.152
Self-efficacy on PLEA-malaria	0.219	0.187	(0.101, 0.336)	0.000
Perceived severity of malaria	0.025	0.019	(− 0.097, 0.146)	0.691
Perceived malaria risk	− 0.010	− 0.009	(− 0.138, 0.118)	0.876
Peer education team spirit	0.102	0.141	(0.027, 0.177)	0.008
Knowledge about malaria	− 0.045	− 0.040	(− 0.146, 0.056)	0.382
Attitude towards malaria preventive measures	− 0.071	− 0.079	(− 0.160, 0.019)	0.120

*ITN* insecticide-treated nets

### Summary of the qualitative results

The qualitative research identified various experiences, perceptions, and perspectives regarding the implementation process and adoption of the school-based PLEA-malaria. The responses were mainly categorized as facilitators and barriers each consisting of several major themes and sub-themes (Table 5). The facilitator consists of six major themes including the PLEA-malaria team-building process, outcome efficacy of PLEA-malaria, presence of the supportive organization in the schools (circle of the student), schools priority, support and follow-up of PLEA-malaria, nature of the school-based SBCC programme, and presence of community-based structures/systems. The barriers have four major themes including low commitments, low malaria threat appraisal, low response efficacy of PLEA-malaria, and planning and implementation gaps.

**Table 5:** Summary of major themes, sub-themes and selected quotations on the facilitators and barriers to the implementation of the school-based of PLEA-malaria, Jimma, Ethiopia, 2017–2019

Major themes	Sub-themes	Selected quotations
Facilitators to the implementation and adoption of the PLEA-malaria		
PLEA team building process and team experiences	Team formation process	*We have already a functional team: The student circle is used to advance the academic performance of our students. The student circle consists of at least six individuals (three male and female each) with one leader and the secretary. So, all team leaders of the students’ circle were nominated for the current training [PLEA-malaria].* A school director, male *The selection of leaders was based on academic performance while considering the gender mix. The students who have good academic performance or higher grade point average in the last semester will be nominated as leader and secretary of the student circle.* Malaria focal teacher, male
	Quality of participation in PLEA	*With student circle… we mean the team members should sit in circle while conducting the peer education or discussion on selected topics. the leader will initiate and present the issue. Then the team members participate and the discussion proceed usually in the form of question and answer.* Female peer educator, grade 8 student *While discussing about malaria among the circle, we all freely present our opinions. One student [the leader] would coordinate the discussion and we all follow him. Our group was eagerly to learn about health issue [malaria] and the process was participatory. Each member reacts to the discussion without any fear because we all are students and importantly friends.* Male, peer educator, grade 6 student
	Team spirit	*During our discussion, we first prepare a topic discussion. We then establish consensus with respects to ideas. We trust each other and free to ask questions or share experiences. Our teacher was in charge of monitoring the process. Every day we conduct peer education at school, we also expected to deliver the information for our families when we are back home.* Female, peer educator, grade 6 student *We have got so many good experiences with working in circle or team. We have learned the effectiveness of working by team compared to working individually. The members have good respects for each other and the leader. We do often decide by consensus.* Male, peer educator, grade 7 student
Outcome efficacy of the programme	Perceived benefits of the program: gain knowledge and skills on malaria	*In our school…, I think students have got adequate knowledge on malaria. we have learned a lot of things. For instance, I myself have got enough knowledge on malaria and how to prevent it. I can also teach my parents about malaria and how to use the mosquito nets.* Female, peer educator, grade 6 student *Teachers acquired not only knowledge about malaria, but also skills needed to guide and implement the PLEA-malaria to sustain the practices* A school director, male
	Perceived benefits of the programme: malaria prevention in community and schools	*As to me I liked the programme. It has great contribution for malaria prevention and control in this area Since, our communities are uneducated about health issue, school students can learn and in-turn teaches their families at large. It would be good if the programme is expanded to reach all schools specially the rural one where many illiterate communities reside.* Female, peer educator, grade 8 student *The programme contributed a lot to malaria control in the community. Look, our ultimate goal was using students to reach parents, neighbours and school friends with malaria preventive information they have learned in school-based peer education. Each team member of student circle will have a home take assignment of teaching parents about malaria. They must report back the parental education activities to schools. There was also a close supervision by the health extension workers in the community.* A school director, male
Presence of organization in the schools facilitated implementation of PLEA	School-based clubs and student circle	*It was very easy for us to implement school-based malaria education activities [PLEA-malaria] since we have active clubs in our school including football club and school health club. We have conducted many school-based communication campaign activities and malaria information dissemination using these clubs. Similarly, regular peer education activities have been conducted in our school by student circle.* A school director, female
Schools priority, support and follow up	school and teachers support	*Effective teaching and learning activities to enable the students would be only possible if the students and the teachers are healthy enough. Maintaining health of the students and the teachers is the first thing we should worry for before education.* School director, male *Students are attending schools for their own mission and teachers are there to teach them. Teachers can bring the students together creating an opportunity for us to approach them and transmit any health message need for the larger community. In this way, we can easily reach the whole community without more challenges.* A HEW, female
Nature of the programme (school-based SBCC)	Participation of frontline stakeholders	*The programme approach was so holistic. It involved everyone including frontline community providers from health, education and teachers. We had regular meeting with these people to discuss about performances, achievement and gaps.* Head, districts education office, male
	Participation of parents	*Parents were not familiar with the programme as they have been reached by their school children. We had been also making a frequent visit to household to monitor the students message delivery practices and parents adoption of the advices.* A HEW, female
	Multiple strategies (training, PLEA, campaigns)	*We have a lot of approaches to implement this malaria programme. First we conduct PE every two weeks. Students are guided to learn from each other and they will be instructed to reach their parents and neighbours with malaria messages. We have frequent school level campaigns to delivery persuasive malaria information.* school director, male
Presence of community-based structures/systems	Connection between HEWs and schools	*As part of health extension packages, malaria prevention education and community behavior change intervention is one of the activities of the HEWs. Malaria prevention campaigns sometimes target schools to reach the rural community using students. So, I believe it would be easy to integrate this programme with routine tasks so that students will be supported.* A director of health department
Barriers to implementation and adoption of the PLEA-malaria		
PE team building process and team experiences	PE members motivation	*The fellow peers were skeptical especially at the early phase of the PE programme, though the problem was resolved gradually by continuous efforts made by the supervisors.* 14 years old and grade 7 students
Low threat appraisal and response efficacy	Low threat appraisal for malaria	*Malaria has seriously affected this community some years ago. Nowadays, it’s getting low. Although, people have some level of fear about this disease, their concerns are getting low and low. The same thing true for our students. Look, previously, the school-dropout due to malaria was high. Now, this number is considerably reduced. But, everyone need to be vigilant.* school director, male
	Low self-efficacy	*Some peer educators feel they are not fit to teach or provide health education on malaria. Thus, in collaboration with people from health offices, we have to provide up to data information on malaria to improve their confidence. We have also guided the students use the information and malaria messages outlined in flip charts distributed for schools.* A school teacher, male
Planning and implementation of PLEA activities	Inappropriate PLEA schedules	*Most often students are forced to sit for PE after quite tiresome class. We all get tired after class, right? Some students do not pay due attention to the discussion. This often led to inadequate discussions.* A male peer educator, grade 8
	lack of uniformity of PE schedule	*In order to balance the time allotted for academic class and for PE activities, we have planned to run the PE every two weeks of Friday.* A school teacher, female
Organization and coordination gaps: fail to harmonize and align roles between schools and health offices	Roles confusion	*Sometimes we get confused about who should do what? Although, the programme was situated in the schools, the question about who should take the lead role was a big challenge. Some health offices received no reports about the programme performance. The schools were also not committed to finding out possible solutions.* A health worker, male
	Teachers priority and staff turnover	*In connection with inadequate training [all school teachers didn’t receive basic training on the programme], they felt they are not appropriate to participate in the programme to guide students in PE activities. the problem arised from the turning over the trained teachers and lack of continuous training and capacity building efforts.* A HEW, female

Accordingly, the participants mentioned that the PLEA-malaria team formation process was easily facilitated by the existing organization in the schools: the circle of students is defined as a group of students consisting of up to six members including one leader and secretary. The circle of students was aimed at quality of education by improving the academic performance in primary schools. Thus, this group was considered to nominate peer educators eligible for the PLEA-malaria. They mentioned that the circle of students was fairly formed with a balanced gender mix and the leaders were selected among higher scorers in the last semester. The participants also reported that there was active participation in peer education activities and positive team spirit, respects and they have enjoyed freely explaining ideas, expressing concerns, and sharing experiences. The student circles also provided structural/networking support through which the PLEA-malaria was effectively implemented and monitored.

The other important factors that were considered facilitators of the implementation and uptake of the programme were the high perceived outcome expectancy PLEA-malaria. The participants perceived that the programme would help them acquire the skill and knowledge on malaria and its prevention methods. They believed the PLEA-malaria had contributed to improving the knowledge and skills of both school teachers and students regarding malaria preventive measures. Moreover, most participants mentioned that the PLEA-malaria is important to advance the community’s and school's malaria preventive practices. The implementation of the PLEA-malaria was facilitated by the existing organizations in the schools such as various school clubs (e.g. health promotion clubs, football clubs). The clubs were used to implement various communication campaigns and message dissemination activities in the schools.

Importantly, schools were concerned about marinating the health of their students and teachers to ensure the quality of the teaching and learning process. They underscored that schools should give priority to health and provide support such as health intervention in order to achieve their academic mission. These concerns might have facilitated the implementation and uptake of the PLEA-malaria that would in turn affect the attitude and acceptance of the programme by peer educators. The participants also described that they liked the programme for the participatory and multiple complementary strategies (e.g. training, PLEA, campaigns) employed that increase exposure to multiple sources of information, reach, and persuasion for the students learning better. While the involvement of frontline stakeholders would provide a supportive learning environment, parental participation can affect the attitudes and acceptance of the school children who serve as health agents for malaria information, and this might, in turn, improve parental social supports. In addition, the existing community-based structures/systems (e.g. the connection between HEWs and schools) facilitated the implementation of these multiple strategies of the programme. The participants discussed that there was a linkage between the HEWs and schools to implement some targeted health interventions such as immunizations and deworming for soil-transmitted helmets.

It was mentioned that the fellow in peer education and school teachers were skeptical especially at the early phase of the programme. The teachers felt they would contribute very little to health/malaria issues as their major responsibility was academic teaching. The other important thing mentioned as barriers to PLEA-malaria was the low threat appraisal of malaria and self-efficacy of the students. This low perception of malaria threat was connected with the declining malaria incidence in the community in recent times. Some participants were also concerned about the knowledge and confidence of the peer in delivering malaria educations to peers and parents.

The other sorts of barriers affecting implementation of the school-based PLEA-malaria were gaps in planning and implementation of the PLEA-malaria activities. They discussed that the inappropriate school-based PLEA-malaria schedules combined with lack of uniformity across schools affected the adoption of the programme. In this regard, the PLEA-malaria session was often performed after tiresome class or just before the students leave for lunch. This affected attention and participation of the team members in the discussion. In addition, there was no fixed schedule for conducting peer education activities and schools were given the responsibility to fix this schedule based on the exiting contexts in the respective schools. As a result, variation or lack of uniformity with peer education schedule was observed both within and across schools that rages from every week to every four weeks. It is clear that the frequency or repetition would greatly affect the dosage of an intervention which in turn affects the outcomes.

Finally, the key informants have also explained they have experienced planning and coordination gaps that resulted from the failure to identify responsibilities align roles and activities between schools, education offices, and health departments. Although the current programme was situated in the schools, the key informants also mentioned that the lack of a clear line of communication between schools and the district's health departments was the major challenge they faced so far. The challenge related to regular reporting or receiving the programme performance reports was also experienced by most of the informants. These gaps were further complicated with the shortage of trained staff to sustain the implementation of the programme. Concerns over the inadequacy of the training on the programme especially for school teachers and the HEWs were described as major challenges. Some participants also mentioned that there was high trained teacher turnover and the lack of continuous provision of training and capacity building efforts as barriers to implementing and sustaining the programme (Table 5).

## Discussion

Enrolling peer educators and key stakeholders, this study examined the implementation and acceptability of the school-based PLEA on malaria prevention and control in primary schools. The study is the first of its kind in Ethiopia that employed a mixed-method to explore the experiences, perceptions, and acceptability of the PLEA-malaria in the targeted schools. Accordingly, the result of the study suggested the measured level of acceptability of the programme by peer educators was considerably high given various facilitators and barriers to its implementation and adoption. Factors such as age in years, number of ITN in the household, frequency of parent-student discussion, parental readiness to attend the discussion, frequency of peer education sessions, self-efficacy, and team spirit were associated with acceptability.

Thus, some previous studies evaluating peer education activities from around the world such as England [37] and Eritrea [14] reported relatively lower scores of acceptability than that of the current study. Successful school-based programmes are often characterized by improved acceptability and feasibility of the intervention which is ultimately is used to sustain it [38, 39]. The collaborative learning nature of such a programme might have been contributed to the improved feasibility [40]. The observed higher perceptions of intervention’s acceptability reported in the current study may be due to many reasons: first, the multi-sectors engagement approach contained within the SBCC framework (i.e. the SBCC was implemented by engaging the education, schools, and health sectors) as this might provide a supportive learning environment. Second, it may be due to the mediating influences of the improvement in the acceptability and feasibility of the malaria SBCC approach among the key stakeholders (lead supervisors) as indicated in the previous study [23]. Third, the improved community/parental acceptance of the malaria education by the students as part of the school-based malaria SBCC might have shaped the attitude of the students towards PLEA-malaria [21]. The finding of this study implies that possibilities or the need for intensifying and replicating such strategies in wider schools for better impacts.

In this study, age was the only socio-demographic factor positively affecting the acceptability of the school-based PLEA-malaria. This goes with the fact that learning and understanding concepts and complex ideas increase with increasing age. The literature showed that learning is facilitated by higher age because younger children do not possess the same ability to learn complex concepts as older children and this might affect the acceptability [41]. The finding implies that considering the age difference while designing and implementing the school-based health education intervention is important. Most importantly, this finding implies such intervention may yield more effects if implemented in the high schools (targeting older students). In the current study, GPA has positively affected the acceptability indicating higher scorers have better acceptability of the programme. The possible reason may be due to the mediating influence of knowledge and self-efficacy towards the PLEA. For instance, self-efficacy was the strongest predictor of acceptability in this study.

Access to ITN or the number of ITN in households is negatively associated with the acceptability towards the PLEA-malaria. The fact that access to the high number of ITN negatively predicted the acceptability of the PLEA-malaria indicates the mediating influence of external factors, beyond the access term, that is rooted in the community settings. This was indicated in qualitative results in which the low community readiness and skepticism about the importance of malaria education, resistance to cooperating during IRS campaigns, and low perception about the efficacy of ITN were mentioned. Furthermore, community contexts such as housing conditions and nature of bedding and inadequacy, the shape of ITN, unavailability of services (IRS and ITNs) were reported as challenges to the uptake of malaria preventive education in the community.

Despite the assumptions that children lack the competence to speak and make decisions about issues that concern them [12], the current study showed the strongest positive relationship between self-efficacy and acceptability of PLEA-malaria. The finding of a study conducted in South Africa reported that self-efficacy positively influenced the readiness to run the peer-education in risky behaviour prevention intervention in schools [42]. Another study reported that peer-educators perceived that their confidence has positively affected the degree and quality of school-based peer education implementation on preventive actions [43]. This implies the school's potential for malaria education and communication using the school children as the social actors and credible sources of malaria. In support of this finding, the participants of the qualitative study believed that the programme had contributed to improving the knowledge and skills of both school teachers and students on malaria. In fact, in one of the similar studies, it was indicated that the students got skills and talents that enabled them to develop persuasive malaria messages (i.e. poems) to influence the local community during campaigns [22].

The frequency of parent-student communication showed a negative association with the acceptability of the PLEA, while the extent of parental readiness to attend home-based malaria education positively affected the acceptability in this study. A study evaluating a school-based health education intervention in Nepal has identified parental attitudes positively affected the intervention’s feasibility while lack of collaboration with parents was mentioned as a challenge [38]. A previous similar study (aimed to evaluate the sustainability of the school-based malaria SBCC) showed the negative effects of the factors such as the community’s reluctance and skepticism of the school-based malaria communication [20]. However, contradicting finding was reported in the other study in which the programme has improved community acceptance of the intervention was reported [21]. Nevertheless, the relationship between the parental readiness to attend the parent-student discussion and acceptability was positive. For this paradoxical association might be due to parents being often preoccupied with daily activities and may lack adequate time to attend the discussion while they still have commitment/readiness to attend it [38].

In this study, the frequency of peer education sessions was positively associated with the acceptability of the PLEA-malaria. The study conducted in Nepal showed that the need to allocate adequate time for the programme in order to bring change in knowledge and practice [38]. In fact, it is clear that the frequency of peer education sessions is influenced by various schools related factors such as the presence of interested teachers and students that positively affected the feasibility of the intervention [38]. However, qualitative research identified that the inappropriate schedules for PLEA combined with the lack of uniformity across and within schools affected the implementation and adoption of the programme. As a result, the peer education session varies ranging from every week to every four weeks. It’s clear that the frequency or repetition would greatly affect the dosage of an intervention which in turn affects the outcomes. Implicitly, the finding suggested the need to consider allocating adequate time for peer education activities while carefully maintaining the balance of schedules for routine academic activities of the schools.

The team spirit in peer education was found to be an important factor that was positively associated with the acceptability of PLEA-malaria. A consistent finding was reported from a study conducted in Eritrea [14] and Australia in which a positive relationship was observed between the peer education programme and students' willingness to share their experience and support each other on health information [26]. Moreover, this finding also proved the fact that "peers depend on peers for information and support", implicating the feasibility of introducing the health-promoting interventions at early adolescent years [44]. The result of the qualitative study identified the presence of positive, friendly, and respectful social relations among members of peer education. The participants reported that they have enjoyed freely explaining ideas, expressing concerns, and sharing experiences. Working in a team was their long-standing experience due to the presence of the existing organizations in the school-called circle of students. The circle of students consists of up to six members including the leader that provided structural support through which the PLEA was implemented. the finding of the current study implies that the importance of building positive social norms in the schools that provide an opportunity for the students to share various personalized experiences and skills to facilitate the ownership of the school-based peer educational interventions.

## Conclusion

The results suggested that the acceptability of the school-based PLEA-malaria was higher implying that it is a promising strategy in promoting malaria prevention and control actions in primary schools by peer educators. Thus, school-based educational and behaviour change interventions that consider demographic factors; (e.g. age, GPA), self-efficacy, access to malaria preventive services (e.g. access to ITN), and social factors, (parental supports, peer education team spirits) would be successful to improving acceptability and ownership of the programme. Moreover, addressing the barriers such as low commitments, threat appraisal, and response efficacy of the students while improving the planning and implementation gaps is required. The relationship about how an improvement in the level of acceptability would in turn influences malaria preventive behaviors among the students should be investigated.

## Supplementary Information

**Additional file 1: Annex 1.** Roles and responsibilities

## Data Availability

The datasets used and analyzed during the current study are available from the corresponding author on reasonable request.
