# Supplementary material for: Acceptability of peer learning and education approach on malaria prevention (PLEA-malaria) through primary schools communities in rural Ethiopia: peer educators’ perspectives
Source: Malar J. 2021 Nov 15;20:437. doi: 10.1186/s12936-021-03965-y (PMC8594237; doi:10.1186/s12936-021-03965-y)
Supplement: Supplementary file 1 — Additional file 1: Annex 1. Roles and responsibilities [file 12936_2021_3965_MOESM1_ESM.docx]

**Annex 1: Roles and responsibilities**

Summary of major roles and responsibility of different actors and stakeholders for the performance of the school-based social and behaviour change communication (SBCC) approach on malaria prevention and control in primary schools in Jimma, Ethiopia, 2019.

| **s.n** | **Actors in the project** | **Descriptions of roles in the project** |
| --- | --- | --- |
| 1 | USAID-Ethiopia | - Financially support the programme - Oversee the project implementation and financial management according to USAID’s rules and regulations - Collects reports and provide feedback on project implementations - Participate in semi-annual and annual review meetings |
| 2 | Jimma University (programmes hosting organization) | - Played a catalyst role - Strengthening capacity of schools and local medias through training, dialogue, supportive supervisions and follow up - Design, develop and provide the IEC/BCC materials - Design and develop IEC/BCC materials and manuals - Develop monitoring and evaluation plan - Ensure integration of the programme into local health system - Regularly monitor the programme - Participate in monthly review meeting and other discussions - Conduct surveys and disseminate results at national and international level. - Also produce scientific reports and journal articles. |
| 3 | School-based malaria SBCC field officers | - Are recruited health staffs to facilitate the SBCC activities in the communities and schools - Coordinate, supervise and monitor of the programme - Participate in training programme - Mobilize schools and religious leaders - Provide communications tools/resources - Own the programme - Collects programmes reports as part of health extension programme (HEP) activities - Participate in review meetings and provide feedback |
| 4 | Zonal health department | - Own the programme through HEP - Coordinate, supervise and monitor of the programme - Attend meetings, sensitization workshops - Collects reports and provide feedback as part of HEP - Supply some communication tools - Scale up best practices and lessons |
| 5 | District Health offices | - Participate in coordination, supervision and monitoring of the programme - Participate in training programmes - Mobilize schools and religious leaders - Provide communications tools/resources - Own the programme - Collects project reports as part of HEP activities - Participate in review meetings and provide feedback - Work closely and coordinate malaria communication activities with district education offices |
| 6 | Health extension workers (HEWs) | - Closely work with schools and contribute in building schools capacities and establish strong linkage with schools. - Provide technical support, supervise, coordinate and monitor the programme - Participate in and lead health education planning by religious leaders and schools - Supplies communication tools for schools and religious leaders - Document/collects, compile and submit reports on project activities as per requirements of HEP reporting system - Participate in monthly meeting at district level - Participate in M&E process |
| 7 | District education offices | - Mobilize schools, students and teachers - Participate in monthly meetings - Support and encourage teachers and students towards working malaria prevention - Participate in malaria prevention campaign - Participate in M&E process - Work closely and coordinate malaria communication activities with district health offices |
| 8 | Primary schools (school directors) | - Collaborate with HEWs, Field offices, Health and education offices - Plan or schedule and coordinate peer education activities - Harmonize team work between teachers and students - Assign teachers for peer education activities - Receive the malaria information, education and communication (IEC) materials (manuals, flip charts, leaflets and posters) from partners and offer it to peer education teams - Supervise the utilization of the IEC materials at peer education session - Plan for school-based small communication campaigns using various school-based clubs (e.g. football matches) - Encourage students take malaria messages for parents - Monitor the parent and students communication (malaria education activities), preparing the parental educational report formats for students - Collect peer education activities reports from focal teachers - Compile and prepare reports on the overall school-based PLEA-malaria activities to districts education offices and districts health departments |
| 9 | Teachers | - Teach students about malaria regularly - Engage, plan, implement and supervise students at peer education activities - Organize and report the peer education and schools malaria communication activities - Demonstrate 4 do-able malaria actions - Teach all teachers about malaria regularly - Facilitate malaria education activities within school and community - Encourage students to teach their families and neighbors - Mobilize students - Participate in M&E process |
| 10 | Students/peer educators | - Select discussion points (based on manuals and other IEC materials) for peer educations - Conduct regular peer education activities - Advice their friends and peers how to prevent malaria - Mobilize school students - Teach/advice their families and neighbors to adopt the 4 do-able actions and eight essential malaria actions. - Participate in malaria prevention activities within school and outside schools such campaign - Participate in community mobilization for prevention and control of malaria - Report the parental and peer education activities (via peer educators) to the immediate teachers (school malaria focal person) |
